# Effects of ROI Placement on PET-Based Assessment of Tumor Response to Therapy

**DOI:** 10.1155/2013/132804

**Published:** 2013-03-07

**Authors:** Mike Sattarivand, Curtis Caldwell, Ian Poon, Hany Soliman, Katherine Mah

**Affiliations:** ^1^Department of Medical Biophysics, University of Toronto, Ontario Cancer Institute, Princess Margaret Hospital, 610 University Avenue, Room 7-411, Toronto, ON, Canada M5G 2M9; ^2^Department of Medical Physics, Odette Cancer Centre, Sunnybrook Health Sciences Center, 2075 Bayview Avenue, Room TG-217, Toronto, ON, Canada M4N 3M5; ^3^Department of Medical Imaging, Faculty of Medicine, University of Toronto, 263 McCaul Street, 4th Floor, Toronto, ON, Canada M5T 1W7; ^4^Department of Radiation Oncology, University of Toronto, FitzGerald Building, 150 College Street, Room 106, Toronto, ON, Canada M5S 3S2; ^5^Department of Radiation Oncology, Odette Cancer Centre, Sunnybrook Health Sciences Center, Toronto, ON, Canada M4N 3M5

## Abstract

*Purpose*. Quantitative PET response assessment during therapy requires regions of interest (ROI). Commonly, a fixed-size ROI is placed at the maximum uptake point in the pretreatment study. For intratreatment, the ROI is placed either at the maximum uptake point (ROI_peak_) or at the same location as the pretreatment ROI (ROI_same_). We have evaluated the effects of the ROI placement on response assessment. *Methods*. PET scans of 15 head and neck cancer patients were used to evaluate the effects of the two ROI methods on response assessment. *Results*. The average intratreatment ROI_peak_ uptake was 13.4% higher than the ROI_same_ uptake (range −14% to 38%). The average relative change in ROI_peak_ uptake was 7.9% lower than ROI_same_ uptake (range −5% to 36%), resulting in ambiguous tumour classification in 19% of the tumours. *Conclusion*. Quantitative PET response assessment using a fixed-size ROI is sensitive the ROI placement. The difference between ROI_peak_ and ROI_same_ could be substantial resulting in ambiguous response assessment. Although the fixed-size ROI is simple to implement, it is also prone to the limitations and should be used with caution. Clinical trial data are necessary to establish reliable thresholds for fixed-size ROI techniques and to evaluate their efficacy for response assessment.

## 1. Introduction

As a powerful molecular imaging tool, positron emission tomography (PET) is increasingly being used for early assessment of tumour response to therapy [13–15]. Typically two sequential PET studies are performed and the tumor standardized uptake value (SUV) in the pre-treatment (Pre-Tx) study is compared to that of the intra-treatment (Intra-Tx) study.

Response assessment using SUVs requires the selection of either a representative tumor voxel or a region of interest (ROI) for quantification. One of the simplest and most common methods of quantifying tumor uptake is to use the single voxel containing the maximum SUV (SUV_max⁡_) [16, 17]. Unfortunately, SUV_max⁡_ values are highly sensitive to image noise and voxel size [18, 19], which leads to uncertainties in quantitative response assessment. Moreover, Krak, et al. [19] reported that SUV_max⁡_ has poor reproducibility compared to estimates of SUV made using ROI methods. As a more robust alternative, an average SUV within a small fixed size ROI has been recommended to provide adequate statistical quality in SUV measurements and to reduce uncertainties in quantitative response assessment [16].

Table 1 lists representative studies [1–12] that have used the fixed-size ROI method for early tumour response assessment. The Pre-Tx ROI is usually centred on the SUV_max⁡_ voxel. However, there are two distinct approaches to the placement of the Intra-Tx ROI. Some studies have centred the Intra-Tx ROI on the SUV_max⁡_ voxel (ROI_peak_), whereas others have placed it at the same location as it was in the Pre-Tx image using anatomical landmarks (ROI_same_).

**Figure 1:**
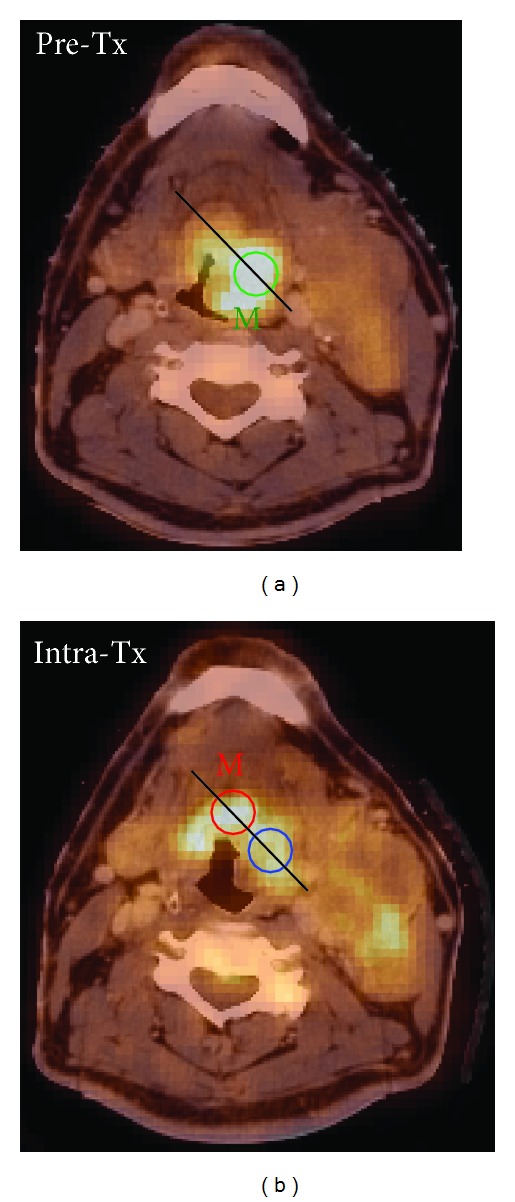
Change in the distribution of FDG uptake during treatment. The PET/CT images of Pre-Tx (a) and Intra-Tx (b) are of a patient with a base of tongue primary tumour. Two circular ROIs of 15 mm diameter are centered at the maximum uptake points on both Pre-Tx (green) and Intra-Tx (red) images denoted by “M.” An additional 15 mm diameter circular ROI is placed on the Intra-TX image (blue) in a position judged to correspond to the same anatomical location as the ROI as in the Pre-Tx. The FDG uptake profiles along the black lines connecting the two Intra-Tx ROIs are shown in Figure 2.

**Figure 2:**
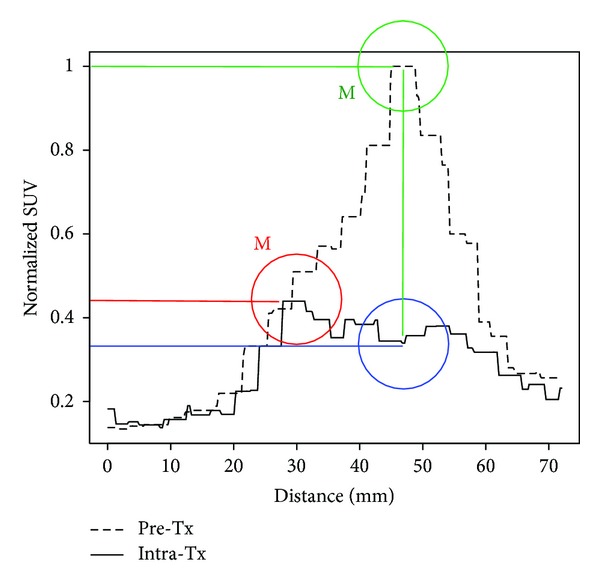
The uptake profile from Figure 1 normalized to Pre-Tx maximum SUV. The distribution of uptake within the tumour has changed during the therapy such that the maximum uptake point along the profile in Pre-Tx corresponds to a local *minimum* uptake point in the Intra-Tx. The maximum uptake point along the profile in Intra-Tx is now in a different location of the tumour.

The distribution of uptake within the tumour may change in response to therapy such that the maximum uptake point in the Intra-Tx study is found at an anatomically different location than it was prior to treatment. This is illustrated in Figures 1 and 2 for a sample head and neck cancer (HNC) patient.  Figure 2 illustrates two quantitative response assessments based on the two different choices of Intra-Tx ROI placement. 

**Figure 3:**
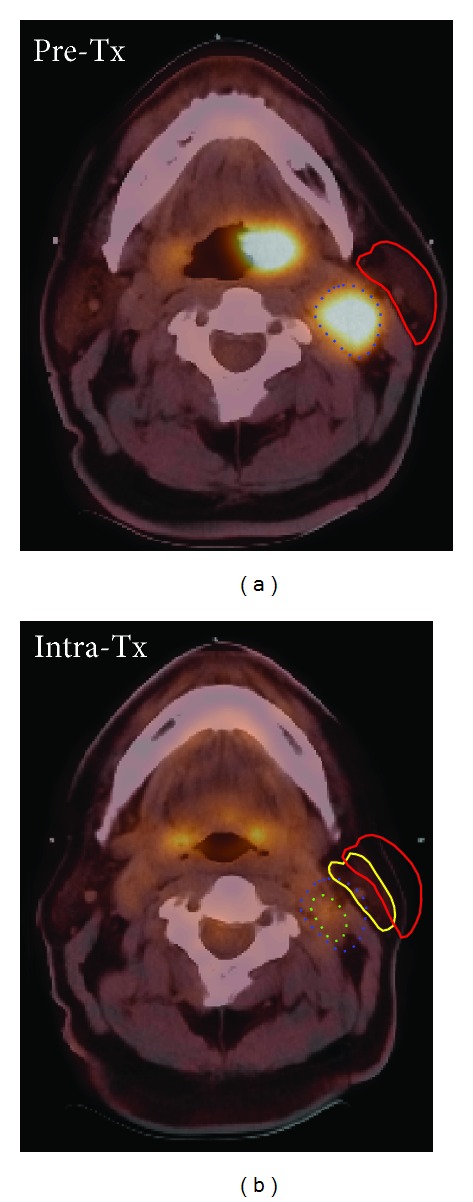
Both tumors and normal tissues may shrink and shift during the treatment. The coregistered PET/CT images of Pre-Tx (a) and Intra-Tx (b) are cross-sectional images of a patient with a primary tumour of the tonsil. The patient's left parotid gland in Intra-Tx (yellow contour) shows volume loss and shift relative to that in Pre-Tx (red contour). Similarly, the gross tumour volume for one nodal disease site in Intra-Tx (dotted green contour) shows volume loss and shift relative to that in Pre-Tx tumour (dotted blue contour).

Using the ROI_same_ method is reasonable if the goal is to evaluate the change in uptake in the same area of the tumour. This method has been recommended by the European Organization for Research and Treatment of Cancer (EORTC) [20]. However, unlike ROI_peak_, tumour response measured by ROI_same_ is prone to uncertainty due to the difficulty in positioning an ROI in the Intra-Tx scan in the exact anatomic location as it was in the Pre-Tx scan. Geometric changes of both tumour and normal tissues may occur during the therapy making it difficult to place an ROI at exactly the same location as it was in the Pre-Tx scan using anatomical landmarks.  Figure 3 shows PET/CT images of a sample HNC patient illustrating the magnitude of typical geometric changes in terms of volume losses and shifts.

Uncertainty in the placement of the Intra-Tx ROI could significantly affect the accuracy of quantitative response assessment. Uncertainties in quantitative response assessment could have significant impact on treatment decisions and clinical outcome. Consequently, we investigated the effects of fixed-size ROI placement on quantitative response assessment. The purpose of this study was twofold: (1) to evaluate quantitative response assessment when Intra-Tx PET images are measured using the ROI_peak_ and ROI_same_ methods; (2) to quantify the geometric changes of both tumour and normal tissues and their impact on quantitative response assessment using the ROI_same_ method.

## 2. Materials and Methods

### 2.1. Design of the Study

Two independent populations (A and B) were used. Population A consisted of 15 patients with a total of 38 gross tumour volumes (GTV) identified by experienced radiation oncologists. Population A was used to compare two quantitative tumour response assessments based on using the ROI_peak_ and ROI_same_ methods. Population B consisted of 10 patients with a total of 33 GTVs identified by experienced radiation oncologists and was used to quantify geometric changes of both tumour and normal tissues during therapy. The impact of these geometric changes on quantitative tumour response assessment was evaluated in population A. Both populations A and B were part of a clinical trial at Sunnybrook Health Sciences Centre (Toronto, Canada) to assess tumour response in patients with advanced HNC. Population B consisted of patients entered in the pilot study which proceeded the main trial while population A consisted of patients entered in the clinical trial itself. 

While populations A and B were very similar, there were some slight differences, primarily in the CT-voxel size used and the average time between the Pre-Tx and Intra-Tx scans. All patients in both groups had locally advanced HNC (stage III or IV) and underwent 6.6 weeks of radical radiotherapy with concurrent chemotherapy. Patients received intensity modulated radiation therapy (IMRT) of 70 Gy in 33 fractions to all GTVs for both primary (GTVp) and involved lymph nodes (GTVn). All patients also received concurrent bolus platinum chemotherapy as tolerated by intravenously injecting 100 mg/m^2^ Cisplatin on days 1, 22, and 43. Patients underwent two sequential FDG-PET/CT scans, one Pre-Tx and one Intra-Tx, both supine in the same position using a thermoplastic radiotherapy immobilization mask. One 18 cm axial field of view (FOV) that covered the head and neck area was used. The PET/CT scanner was the GEMINI System (Philips Medical System, Cleveland, Ohio). Prior to the PET/CT scans, patients were injected with 5 MBq of FDG per kg. Patients heavier than 75 kg were injected with a fixed dose of 370 MBq of FDG. PET images were reconstructed using a 3-Dimensional Row-Action Maximum Likelihood Algorithm (3D-RAMLA) and corrected for attenuation using CT. In order to register the Intra-Tx CT to the Pre-Tx CT images, a Chamfer matching algorithm [21] based on bony structures was implemented in house using the Interactive Data Language (IDL) Ver. 6.4 (Research Systems Inc., Boulder, CO). The algorithm used 3D rigid body with rotation and translation but no scaling. An IDL program was also developed in house to simultaneously display the registered Pre-Tx and Intra-Tx PET/CT images, to contour ROIs, and to read SUV values. PET images were interpolated to match the voxel sizes of CT images. All statistical analyses were performed using the public domain package “R” (http://www.r-project.org/).

### 2.2. Population A

Pre-Tx FDG PET/CT scans were performed 14 ± 4 days (range, 8–22) prior to the start of the treatment. Intra-Tx FDG PET/CT scans were performed 16 ± 2 days (range, 11–20) after the first treatment day. The CT-voxel size was 0.59 × 0.59 × 1.60 mm^3^ and the CT FOV was 300 × 300 × 210 mm^3^ in lateral, anterior-posterior, and superior-inferior directions, respectively. The PET voxel size was 2 × 2 × 2 mm^3^ and the PET FOV was 576 × 576 × 180 mm^3^ in lateral, anterior-posterior, and superior-inferior directions, respectively. PET images were acquired 50 minutes after injection for 2.5 minutes. The Pre-Tx and Intra-Tx PET postinjection acquisition times were matched within 5 minutes.

The SUVs were normalized to the patients' body weight. For each GTV, ROI_peak_ (a circular ROI of 15 mm diameter) was placed on a single transaxial slice centered at the maximum FDG uptake point in both Pre-Tx and Intra-Tx images. For each GTV, ROI_same_ (a circular ROI of 15 mm diameter) was also placed on a single transaxial slice at the location of the Intra-Tx image that corresponded to the same physical location as the Pre-Tx max-point ROI. A dual-board certified, nuclear medicine/radiology physician positioned ROI_same_ based on anatomical landmarks. Thus, each GTV had two Intra-Tx ROIs. The distance between the centers of these two ROIs was measured in 3D geometry.

On the same transaxial slice where ROI_same_ was located, the Intra-Tx GTV size was measured by averaging the anterior-posterior and lateral extents of an oncologist drawn GTV. In order to reduce errors in FDG uptake from partial volume effects, only Intra-Tx GTVs larger than 15 mm were subsequently analyzed, reducing the total number of GTVs available for analysis from 38 to 26.

Tumour response assessments were obtained using two different methods, called ΔSUV_peak_ and ΔSUV_same_, by calculating the relative change in tumour uptake:

(1) $$\begin{array}{c} \Delta {\text{SUV}}_{\text{peak}}=1-\frac{\text{IntraTx}\;{\text{SUV}}_{\text{peak}}}{\text{PreTx}\;{\text{SUV}}_{\text{peak}}},\\ \Delta {\text{SUV}}_{\text{same}}=1-\frac{\text{IntraTx}\;{\text{SUV}}_{\text{same}}}{\text{PreTx}\;{\text{SUV}}_{\text{same}}}, \end{array}$$

where SUV_peak_ is the mean SUV within ROI_peak_ in either Pre-Tx or Intra-Tx PET images. SUV_same_ is the mean SUV within the ROI_same_ in the Intra-Tx PET image. A positive value for ΔSUV_peak_ or ΔSUV_same_ indicates a decrease in uptake and a negative value indicates an increase in uptake.

In order to determine how uncertainties in positioning ROI_same_ due to geometric changes may impact ΔSUV_same_ values, the original ROI_same_ was systematically shifted in a 3D grid geometry up to 25 mm in three orthogonal directions. The sampling spaces of the grid were 1.17, 1.17, and 1.60 mm in the lateral, anterior-posterior, and superior-inferior directions, respectively. For each point in the grid SUV_same_ was determined. This data set was sorted based on the distance of the shifted ROI_same_ to the original ROI_same_. For each GTV, SUV_same_ was calculated and plotted as a function of this distance (i.e., positioning error). Each plot was normalized to the SUV_same_ of the original ROI_same_. This normalization makes the *y*-axes represented also normalized (1 − ΔSUV_same_). Plots of normalized SUV_same_ were averaged over 16 GTVs with Intra-Tx size smaller than 30 mm or 10 GTVs with Intra-Tx size larger than 30 mm. The arbitrary 30 mm threshold (twice the ROI size) was chosen to emphasize the effects due primarily to tumour uptake heterogeneity versus the effects due primarily to the partial volume effect. Tumour uptake heterogeneity was expected to have greater impact in large GTVs (>30 mm) and the partial volume effect was expected to have greater impact in small GTVs (<30 mm).

### 2.3. Population B

Pre-Tx FDG PET/CT scans were performed 17 ± 5 days (range, 13–28) prior to the start of the treatment. Intra-Tx FDG PET/CT scans were performed 33 ± 4 days (range, 28–40) after the first treatment day. The CT-voxel size was 1.17 × 1.17 × 6.5 mm^3^ and the CT FOV was 600 × 600 × 208 mm^3^ in the lateral, anterior-posterior, and superior-inferior directions, respectively. The PET voxel size was 2 × 2 × 2 mm^3^ and the PET FOV was 576 × 576 × 180 mm^3^ in the lateral, anterior-posterior, and superior-inferior directions, respectively. 

GTVs were contoured manually by an oncologist experienced in treatment of HNC. All the GTVs were contoured on CT images guided by coregistered PET images. Noncoregistered diagnostic MR images were available to aid contouring in all patients except one where no MRI study was performed. Radiology reports on both PET/CT and MRI studies were also used to aid in contouring.

Geometric changes of the GTVs and normal tissues during treatment were thought to be potentially important in influencing the accuracy of placement of an ROI for quantitative tumour response assessment. In addition to GTVs, geometric changes of some normal tissues were also quantified. While the geometric shifts in tumours, not normal tissues, were of primary interest, the uncertainty in estimating geometric shifts in GTVs was greater than the uncertainty in estimating shifts in other structures, simply due to the difficulty in accurately delineating the GTV after treatment. Thus, the geometric shifts in normal tissues were used as surrogate measures of possible shifts in GTVs. Ten normal tissues were contoured on both Pre-Tx and Intra-Tx CT images for each patient. These normal tissues included the C2 vertebral body, mandible, hyoid, spinal cord, right and left sternocleidomastoid muscles, right and left parotid glands, and right and left submandibular glands. All normal tissues were contoured using consistent window and level settings under the guidance of an experienced oncologist. The most inferior extent for contouring the spinal cord and the sternocleidomastoid muscles was the most superior aspect of the apex of the lung. The most superior extent of the spinal cord was chosen to correspond to the most superior extent of the C2 vertebral body. Mandible and parotid contours were excluded from one patient since the scan did not include the entire organs in the superior direction.

Using both Pre-Tx and Intra-Tx contours for normal tissues and GTVs, an IDL program was developed in house to quantify the geometric changes by calculating

1. percentage volume changes, that is, Intra-Tx volume relative to Pre-Tx volume,
2. shift of center Of mass (COM), that is, Intra-Tx COM relative to Pre-Tx COM. The shifts were calculated as a shift vector in a 3D geometry and the reported values are the absolute values of these vectors.

## 3. Results

Patient characteristics for both populations are listed in Table 2.

### 3.1. Population A

**Figure 4:**
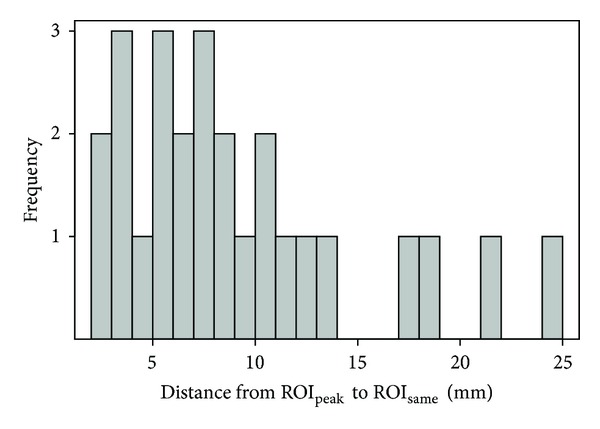
Histogram of distances between the centers of the two Intra-Tx ROIs.

The mean Intra-Tx GTV size (i.e., average of the anterior-posterior and lateral extents) was 25.7 ± 8.9 mm (range, 15.1–46.5). A histogram of the distances between the centers of the two Intra-Tx ROIs for each GTV is shown in Figure 4. This histogram shows that the Intra-Tx maximum uptake point does not normally correspond to the same physical location as that for Pre-Tx. The median distance between the centers of the two Intra-Tx ROIs was 7.4 mm. The two Intra-Tx ROIs were on the same transaxial slice in only 8% of the cases (in 2 out of 26 GTVs). 

**Figure 5:**
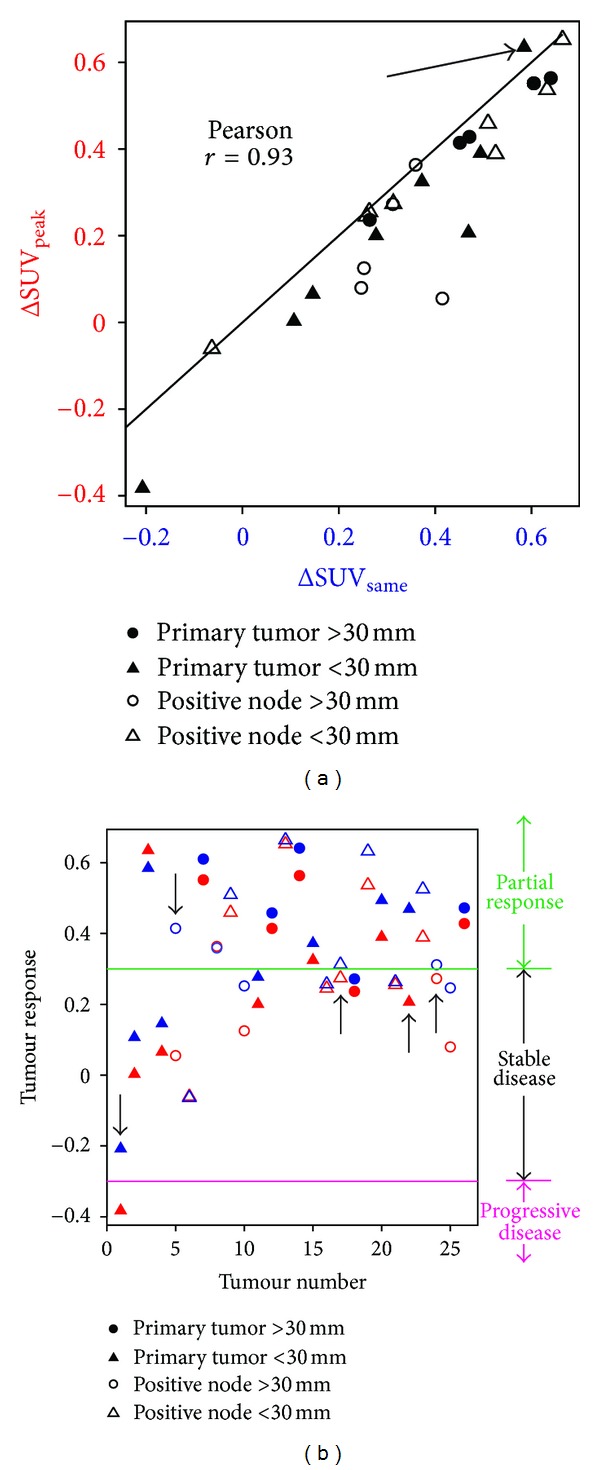
Comparison between the two quantitative tumour response measurements when two different Intra = Tx ROI methods were used. Plot (a) is a scatter plot of the two methods. The solid line in this graph is the unity line where ΔSUV_peak_ = ΔSUV_same_. For most tumours  ΔSUV_peak_ ≤ ΔSUV_same_. An outlier is identified by the oblique arrow above the unity line where ΔSUV_peak_ > ΔSUV_same_. In plot (b), the tumour response on *y*-axis is plotted for all 26 tumours on *x*-axis. Thresholds of ±30% as defined by PERCIST were applied to separate individual tumours to different categories using either ΔSUV_peak_ (red) or ΔSUV_same_ (blue). 19% of the tumours (5 out of 26) were ambiguously classified as shown by vertical arrows.

Figure 5(a) shows a scatter plot comparing quantitative tumour response assessments using the ROI_peak_ and ROI_same_ approaches. A high two-sided Pearson correlation coefficient was found between ΔSUV_peak_ and ΔSUV_same_ (*r* = 0.93, *p* = 7*e* − 12) for all GTVs. Similarly, the *r* value between Intra-Tx SUV_peak_ and ΔSUV_same_ was 0.92, *p* = 5*e* − 11 for all GTVs.

As expected, Intra-Tx SUV_peak_ had a higher value than SUV_same_ for most GTVs, resulting in a lower value for ΔSUV_peak_ compared to ΔSUV_same_ as seen in Figure 5(a). On average, SUV_peak_ was 13.4% higher than SUV_same_ (range −14% to 38%) and ΔSUV_peak_ was 7.9% lower than ΔSUV_same_ (range −5% to 36%). One unusual case, identified by the oblique arrow in Figure 5(a), is an example where the ΔSUV_peak_ was 5.3% higher than  ΔSUV_same_. In this case, the ROI_peak_ region placed centred on the peak voxel in the Intra-Tx scan actually had a lower average uptake than the ROI_same_ region. GTVs in Figure 5 are coded for primary versus nodal mass as well as for large (>30 mm) versus small (<30 mm) GTVs. No statistically significance difference was found between ΔSUV_peak_ and ΔSUV_same_ on the basis of GTV size (large versus small) or type (primary versus node).

Figure 5(b) shows classification of individual tumours based on the PET Response Criteria in Solid Tumors (PERCIST) [16] using either ΔSUV_peak_ or ΔSUV_same_. The PERCIST thresholds of ±30% were applied to classify individual tumours into three categories of partial response, stable disease, and progressive disease. In 19% (5 out of 26) of the tumours this resulted in ambiguous tumour classification depending on the ROI method as shown by the arrows.

**Figure 6:**
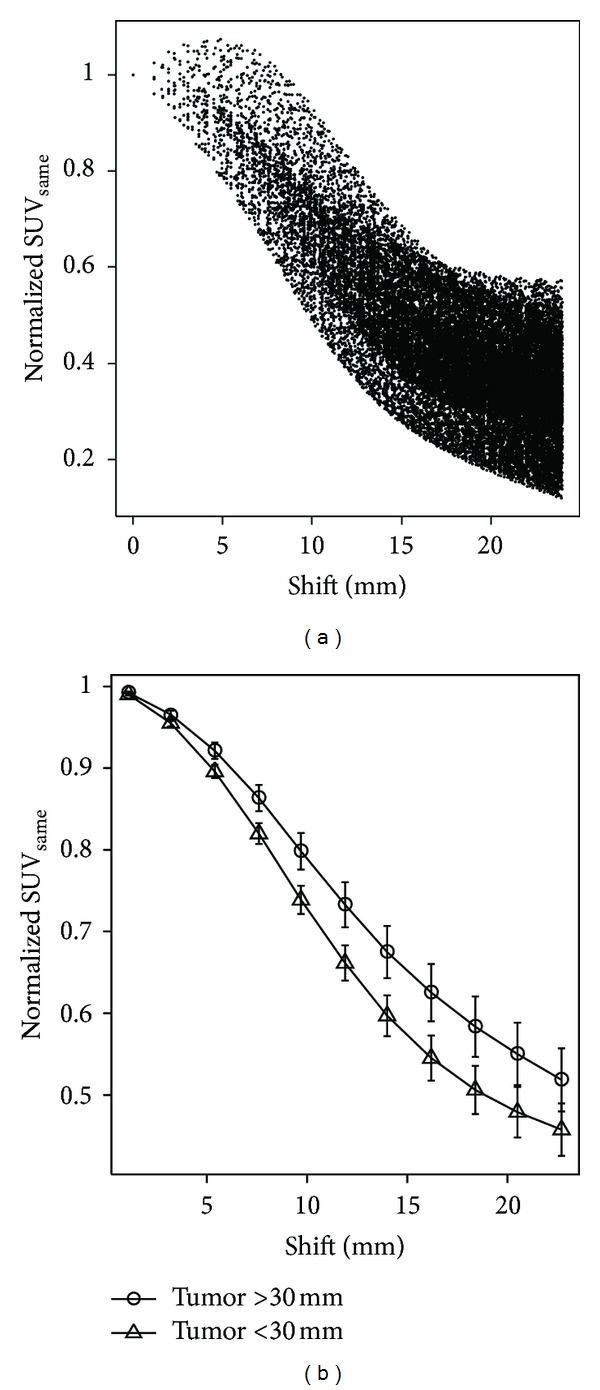
Plots showing how uncertainties in positioning ROI_same_ impact tumor response assessment measured by ΔSUV_same_. Data for a sample tumor (a) and the average data for all tumors (b) are shown. The error bars represent standard errors.

Figure 6(a) shows an example plot for a tumour, demonstrating how uncertainties in positioning ROI_same_ may impact ΔSUV_same_ values. This plot shows that positioning the ROI_same_ a few millimeters away may decrease or increase ΔSUV_same_ depending on whether ROI_same_ is moving towards the maximum uptake point or is moving away from it. However, by moving a few centimeters away, the points eventually start to drop since the ROI_same_ is sampling the background normal tissue uptake. Individual plots such as Figure 6(a) were averaged for all GTVs. The results are shown as two curves in Figure 6(b) based on Intra-Tx GTV size bigger or smaller than 30 mm. A statistically significant difference between the two curves at the 95% confidence level was found to be between 5.4 mm and 16.2 mm.

### 3.2. Population B

**Figure 7:**
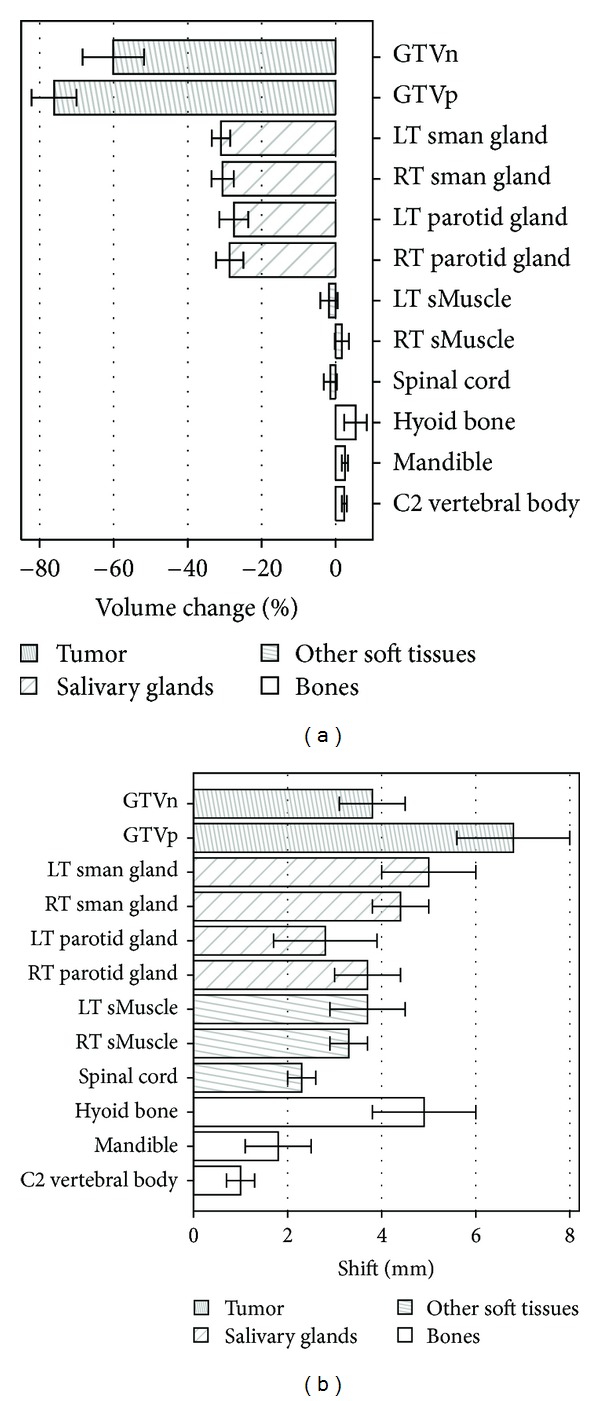
Geometric changes due to therapy for GTV and normal tissues characterized by percentage volume changes (a) and shifts (b). The bars show the median values and the error bars show the standard errors. GTVp: gross tumor volume (primary), GTVn: involved lymph node, LT: left, RT: right, sman: submandibular, sMuscle: sternocleidomastoid muscle.

A total of 97 normal tissue regions were contoured in 10 patients in both Pre-Tx and Intra-Tx.  Figure 7 shows geometric changes due to therapy characterized in terms of percentage volume changes and COM shifts.  Figure 7(a) shows the percentage volume changes for all GTVs and normal tissues. Negative volume changes indicate a loss of volume during therapy. Both GTVp and GTVn showed significant volume losses with median values of 76.1% and 60.1%, respectively. The median volume loss for all GTVs was 67.2% (range, 8.4–96.9%).

For normal tissues, significant volume losses were only found for the salivary glands. Median volume losses were 28.1% (range, 7.3–45.6%) for all parotid glands and 31.0% (range, 13.3–48.7%) for all submandibular glands. Other soft tissues (i.e., sternocleidomastoid muscles and spinal cord) and bones did not show significant volume losses.

Figure 7(b) shows COM shifts in GTVs and normal tissues. The median shift for all GTVs was 5.9 mm and the 95% CI range was 4.4–7.6 mm. The C2 vertebral body showed the smallest shift with a median of 1.0 mm. Right and Left parotid glands showed median shift values of 3.7 mm and 2.8 mm, respectively. The median shifts in medial directions for right and left parotid glands were 1.4 mm and 2.5 mm, respectively.

## 4. Discussion

Geometric changes during therapy can be expected to influence the accuracy with which an expert can place the tumour ROI and thus could affect tumour response assessment using the ROI_same_ approach. Our results in Figure 7 are similar to those reported earlier [22, 23]. We found that the 95% CI for GTV COM shift is between 4.4 and 7.6 mm. This range may represent the upper range of uncertainty in placing the Intra-Tx tumour ROI at the same location as the Pre-Tx tumour ROI. However in practice, attempts are made to correct for the geometric changes to some extent using anatomical landmarks. Moreover, in our study, population A had earlier Intra-Tx scans than population B. Due to these two factors, we expect that the uncertainties in placing the Intra-Tx tumour ROI have a smaller range than the 95% CI shift, possibly in the 0–5 mm range. Based on Figure 6(b), the impact of this uncertainty can be expected to be less than 10% on the measure of tumour response.

The placement of the fixed size ROI could have a significant effect on PET quantification for tumour response assessment. In this study, we found that ΔSUV_peak_ was 7.9% lower than ΔSUV_same_ on average, and difference was up to 36%. This degree of difference leads to different response assessment using PERCIST [16], resulting in overall 19% (5 out of 26) ambiguous tumour response assessment (Figure 5(b)). This finding underscores the need for an optimized PET quantification method in individual patients using a consistent and standard ROI for an accurate response assessment. A small fixed size ROI placed on a single slice is a simplistic approach to sample tumour uptake.  Figure 2 demonstrates that the change in heterogeneity within the tumour due to treatment could be significant. This indicates the disadvantage of PET quantification for response assessment using a small fixed size ROI [24]. 

With ΔSUV_peak_ one may risk overestimating response to treatment compared to ΔSUV_same_. This difference directly results from the fact that ΔSUV_peak_ was on average 13.4% higher than SUV_same_ since it was centered at the maximum uptake point. Occasionally, SUV_peak_ may be smaller than SUV_same_. The outlier in Figure 5(a) corresponds to a situation where the central pixel of ROI_peak_ has a high uptake but its surrounding pixels have a lower uptake than the pixels within ROI_same_. Noisy PET images or high intratumour uptake heterogeneity might cause such a situation.

Considering the typical response thresholds which have been used to separate responding patients from nonresponding patients (last column in Table 1), the difference of 7.9% (and up to 36%) between the two ROI methods could be clinically significant. 

Many recent studies on early tumour response assessment have used the single-voxel based SUV_max⁡_ method, while the new recommendation favors a fixed size ROI as a more robust alternative to reduce uncertainties due to noise [16]. The placement of the fixed size ROI in Intra-Tx, whether ROI_peak_ or ROI_same_ as per EORTC recommendations [20], could lead to significant uncertainties in response assessment. Thus, more studies are required to determine if either of these simple, fixed size ROI approaches are useful in assessing treatment response. 

We found that the two ROI methods gave rise to highly correlated (*r* = 0.93) response assessments (Figure 5(a)). This high correlation is a direct result of high correlation (*r* = 0.92) between the SUV values of the two Intra-Tx ROI methods. This suggests that the higher uptake in ROI_peak_ also means potentially higher uptake in ROI_same_. ROI_same_ in general was sampling a different part of the tumour at some distance away from ROI_peak_ (Figure 4). Part (but not all) of this correlation can also be explained by overlap of the two Intra-Tx ROIs (both 15 mm in diameter). In our patients, the two ROIs were in the same slice in only 8% of the GTVs. It is unsurprising that the pattern of tumour uptake could be considerably changed in response to therapy. Even without therapy, the pattern of uptake over time may alter as the tumour grows. 

The EORTC [20] recommends placing the Intra-Tx ROI at the same anatomical location as the Pre-Tx ROI in order to sample the same area. This is a reasonable approach, that is, to evaluate the same location before and after some therapeutic intervention. It does not seem as intuitively reasonable to use the ROI_peak_ approach, which could mean comparing two anatomically distinct parts of the tumour before and after therapy. However, in a limited number of patients, we found that the two ROI methods were highly correlated (*r* = 0.93). This suggests that the two response assessment methods would likely have a similar accuracy in terms of differentiating responders versus nonresponders, although with different optimal response threshold values. In order to determine if a simple fixed ROI-based method has true utility for assessing response, substantial clinical trial data including patient outcomes is required. Such trial data could also be used to establish if there are threshold levels for ROI-based techniques that could reliably separate responders from nonresponders for each disease site and given treatment type. 

## 5. Conclusion

PET quantification for assessing treatment response using a fixed size ROI is sensitive to the placement of the ROI within the tumour. The difference between the current recommendations favoring ROI_peak_ (over ROI_max⁡_) and earlier recommendations using ROI_same_ could be substantial (36%) resulting in ambiguous treatment response assessment (19%). Methods making use of such small ROIs have the advantage of being relatively simple to implement while still providing improved statistical properties versus the SUV_max⁡_ single voxel method. However, simplicity is not always an advantage and the use of a small fixed size ROI for tumour response assessment should be approached with caution in heterogeneous tumours. Clinical trials are necessary to compare the efficacy of a fixed size ROI over ROI_max⁡_ and establish a reliable threshold in a given cancer site.

## Figures and Tables

**Table 1:** A summary of previous Intra-Tx tumour response assessment studies that used the Fixed size ROI method.

Study	*n*	Site	Pre-Tx ROI	Intra-Tx ROI	Res. Thr
Schelling et al. (2000) [1]	22	Breast	Fixed size, circular 15 mm at max	Fixed size, circular 15 mm at max	55%
Weber et al. (2003) [2]	57	Lung	Fixed size, circular 15 mm at max	Fixed size, circular 15 mm at max	20%
Avril et al. (2005) [3]	33	Ovarian	Fixed size, circular 15 mm at max	Fixed size, circular 15 mm at max	20%
Brun et al. (2002) [4]	47	Head and neck	Fixed size, square (4 or 9 pixels) at max	Fixed size, square (4 or 9 pixels) at max	Median
Rousseau et al. (2006) [5]	64	Breast	Fixed size, 5 to 10 mm at max	Fixed size, 5 to 10 mm at max	40%
Maisonobe et al. (2013) [6]	40	Colorectal	Fixed size, 3 × 3 × 3 voxels at max	Fixed size, 3 × 3 × 3 voxels at max	N/Sp
Ott et al. (2006) [7]	65	EG junction	Fixed size, circular 15 mm at max	Fixed size, circular 15 mm at the same position using landmark	35%
Weber et al. (2001) [8]	40	EG junction	Fixed size, circular 15 mm at max	Fixed size, circular 15 mm at the same position using landmark	35%
Wieder et al. (2007) [9]	24	EG junction	Fixed size, circular 15 mm at max	Fixed size, circular 15 mm at the same position using landmark	35%
Ott et al. (2003) [10]	44	Gastric	Fixed size, circular 15 mm at max	Fixed size, circular 15 mm at the same position using landmark	35%
Wieder et al. (2004) [11]	38	Esophagus	Fixed size, circular 15 mm at max	N/Sp	30%
Schwarz et al. (2005) [12]	11	Breast	Fixed size, circular (size N/Sp) manually placed	Fixed size, circular (size N/Sp) manually placed	20%

*n*: number of patients, Pre-Tx: pretreatment, Intra-Tx: intratreatment, ROI: region of interest, Res. Thr.: response threshold, EG: esophagogastric, N/Sp: not specified.

**Table 2:** Patient characteristics.

	Population A	Population B
No. of patients	15	10
Sex (F, M)	3 F, 12 M	1 F, 9 M
Age		
Mean age ± SD	58.3 ± 5.7 yr	58.7 ± 11.6 yr
Age range	49–68 yr	42–79 yr
Clinical stage		
Stage III	5	1
Stage IV	10	9
Total no. of GTV	38	33
GTVp	15	10
GTVn	23	23
Site		
Tongue	5	4
Tonsil	3	3
Hypopharynx	5	1
Larynx	2	1
Paranasal sinus	0	1

F: female, M: male, SD: standard deviation, GTV: gross tumour volume, GTVp: primary tumour, GTVn: involved lymph node.
